# Supplementary material for: Does the pressure to fill journal quotas bias evaluation?: Evidence from publication delays and rejection rates
Source: PLoS One. 2020 Aug 11;15(8):e0236927. doi: 10.1371/journal.pone.0236927 (PMC7418967; doi:10.1371/journal.pone.0236927)
Supplement: S1 Appendix — (PDF) [file pone.0236927.s001.pdf]

Appendix.

Proof of Proposition

We introduce some notations. Let dN_t denote the arrival of paper at t (i.e., $dN_t = 1$ ($dN_t = 0$) represents that a paper arrives (does not arrive) at t). Let $V(k,t)$ denote the value function of the editor who already accepted k papers, i.e., $V(k,t)$ is the maximum expected quality of remaining $(K-k)$ papers under the editor's optimal policy. For boundary conditions, it is straightforward to show that

$$V(K,t) = 0 \text{ for all } t \text{ and } V(k,T) = 0 \text{ for all } k. \tag{1}$$

Noting that after the editor accepts $(k+1)$-th paper at t, the value function shifts from $V(k,t)$ to $V(k+1,t)$, and the difference should be compensated by the threshold:

$$\underline{q}(k,t) = V(k,t) - V(k+1,t). \tag{2}$$

We can restrict that q is distributed smoothly over $[0,\infty)$.[1] Hence, we have the boundary condition of $\underline{q}(K-1,T) = 0$, which in conjunction with (1) and (2) yields that

$$\underline{q}(k,T) = 0 \text{ for all } k. \tag{3}$$

It is convenient to use $g(\underline{q})$, defined as follows:

$$g(\underline{q}) = \mathbb{E}\left[\left(q - \underline{q}\right)\mathbf{1}\left(q > \underline{q}\right)\right]. \tag{4}$$

Note the the distribution of q is smooth, $g(\underline{q})$ has the following property:

$$g(\underline{q}) > 0 \text{ and } \frac{\partial g(\underline{q})}{\partial \underline{q}} < 0. \tag{5}$$

Next, we examine the dynamics of $q(k,t)$. The value function satisfies the following:

$$V(k,t) = \mathbb{E}\left[(q + V(k+1,t+dt))\mathbf{1}\left(q > \underline{q}(k,t)\right)dN_t + V(k,t+dt)\left(1 - \mathbf{1}\left(q > \underline{q}(k,t)\right)dN_t\right)\right],$$

[1] Since the editor wants to maximize the overall quality of accepted papers, the editor can treat papers with negative qualities as non-arrival of papers.

which in conjunction with (4) yields that

$$\frac{\partial V(k,t)}{\partial t} = -\lambda g\left(\underline{q}(k,t)\right). \tag{6}$$

From (2), we have

$$\frac{\partial V(k,t)}{\partial t} = \frac{\partial \underline{q}(k,t)}{\partial t} + \frac{\partial V(k+1,t)}{\partial t},$$

which along with (6) implies that

$$\frac{\partial \underline{q}(k,t)}{\partial t} = -\lambda g\left(\underline{q}(k,t)\right) + \lambda g\left(\underline{q}(k+1,t)\right) \text{ for } k < K-1 \tag{7}$$

$$\frac{\partial \underline{q}(K-1,t)}{\partial t} = -\lambda g\left(\underline{q}(K-1,t)\right) \le 0 \tag{8}$$

Now, we are ready to show $\underline{q}(k,t) \le \underline{q}(k+1,t)$. We use mathematical induction. First, the followings are satisfied when $k+1 = K-1$:

$$\underline{q}(k+1,T) = 0 \tag{9}$$

$$\frac{\partial \underline{q}(k+1,t)}{\partial t} \le 0 \tag{10}$$

$$\underline{q}(k+1,T) - \underline{q}(k,T) = 0, \tag{11}$$

where the first and last conditions are from (3) and the second is from (8).

Second, we show that under the conditions of (9)-(11), it holds that $\underline{q}(k,t) \le \underline{q}(k+1,t)$. Define $\Delta_k(s) = q(k+1,T-s) - q(k,T-s)$ for $s \in [0,T]$. Note that $\Delta_k(0) \ge 0$ from (11). Fix $s_0 \ge 0$. Assume that $\Delta_n(s_0) \ge 0$. Then, it holds that

$$\begin{aligned}\frac{\partial \Delta_k}{\partial s}|_{s=s_0} &= -\frac{\partial \underline{q}(k+1,T-s)}{\partial(T-s)} + \frac{\partial \underline{q}(k,T-s)}{\partial(T-s)} \ge \frac{\partial q(k,T-s)}{\partial(T-s)}|_{s=s_0} \\ &= -\lambda g\left(q(k,T-s_0)\right) + \lambda g\left(q(k+1,T-s_0)\right) \\ &= -\lambda g\left(q(k+1,T-s_0) - \Delta_k(s_0)\right) + \lambda g\left(q(k+1,T-s_0)\right),\end{aligned}$$

where the first inequality is from (10), the second equality is from (8) and the last equality is from

the definition of $\Delta_k(s)$. The above inequality shows that when $\Delta_k(s) \downarrow 0$, the lower bound of $\frac{\partial \Delta_k}{\partial s}$ approaches to zero, preventing $\Delta_k(s)$ from going below zero. Hence, $\Delta_k(s) \geq 0$ for $s \in [0, T]$, implying that

$$\underline{q}(k,t) \leq \underline{q}(k+1,t). \tag{12}$$

Lastly, we complete the induction by confirming the following:

$$\begin{aligned} \underline{q}(k,T) &= 0 \\ \frac{\partial \underline{q}(k,t)}{\partial t} &\leq 0 \\ \underline{q}(k,T) - \underline{q}(k-1,T) &= 0, \end{aligned}$$

where the first and last conditions are from (3). The second inequality is obtained by applying (5) and (12) into (7). Hence, from mathmatical induction, it holds that $\underline{q}(k,t) \leq \underline{q}(k+1,t)$ for all k. This completes the proof of the proposition. □

□
