# Supplementary material for: Does the pressure to fill journal quotas bias evaluation?: Evidence from publication delays and rejection rates
Source: PLoS One. 2020 Aug 11;15(8):e0236927. doi: 10.1371/journal.pone.0236927 (PMC7418967; doi:10.1371/journal.pone.0236927)
Supplement: Attachment — Submitted filename: Response_v1.docx [file pone.0236927.s002.docx]

**Response to referee comments**

**Reviewer 1**

*As I understand it, the data are very coarse, and are measured on an annual basis. It would be much better to have data on a monthly basis, but I understand that might not be available. What would want to try to recover is the editor’s decision at a specific point in time t (i.e., what is the backlog at this time when he/she is evaluating a group of papers?). Since editors don’t make decisions at one point in time in the year, averaging across the year might generate some bias. I think the bias is probably going to attenuate the effect, but the authors need to more copiously and cogently deal with this issue of measurement error. This is particularly because the effect sizes seem very small to me (keep in mind these are log-log models, so 0.8% does not mean 0.8 percentage points). It could reflect going from 10% acceptance rate to 9.9%.*

First of all, we appreciate your constructive comment. We agree with your concern about attenuation bias, given the small effect size. Unfortunately, we do not have monthly data, but we can address your concern on the attenuation bias, thus.

Starting with the scenario that the true data generating process is given by

$\log\left( {RejRate}_{m} \right)=a+b\log\left( {PubDelay}_{m-1} \right)+e_{m}$	(1)

we fit the model using the observed annual data, not the monthly data, as follows:

$\log\left( {RejRate}_{y} \right)=\alpha+\beta\log\left( {PubDelay}_{y-1} \right)+e_{y}$	(2)

where

$$\log\left( {RejRate}_{y} \right)=\frac{1}{12}\sum_{m\in y} log({RejRate}_{m})$$

$$\log\left( {PubDelay}_{y} \right)=\frac{1}{12}\sum_{m\in y} log({PubDelay}_{m})$$

Then, we examine the following question: what would be (a, b) in the true data generating process of (1) to observe $(\alpha, \beta)$ of (-0.394, 0.084) in the empirically fitted model in Column 2 of Table 3.

To answer this question, we perform a simulation exercise. In particular, we simulate the monthly data by setting (1) as follows:

$$\log\left( {RejRate}_{m} \right)=-.45+0.15\log\left( {PubDelay}_{m-1} \right)+0.1 \mathcal{N}(0, 1)$$

Also, we simulate the monthly publication delay as follows:

$$\log\left( {PubDelay}_{m} \right)=1.708+0.93(\log\left( {PubDelay}_{m-1} \right)-1.708)+0.1 \mathcal{N}(0, 1)$$

where 1.708 is the mean of the log of PubDelay and 0.93 is chosen to fit the annual frequency serial correlation of 0.6. After we simulate the monthly process above, we convert the data into 500 annual observations as in our empirical exercise and fit the annual data to (2). The following figure is the histogram of fitted $(\alpha, \beta)$ from 10,000 repetitions. On average, we obtain 0.87 for the level of $\beta$, similar to our empirical observation. That is, as the referee pointed out, we find a substantial attenuation, roughly a 40% reduction, when measuring the quantitative relationship between rejection rates and publication delay.

**Figure R1:** Histogram of $(\alpha, \beta)$ fitting (2) with simulated 500 annual data out of 10,000 repetitions

*Further, some of the author decisions seemed sloppy, such as only using one randomly selected paper per year to estimate review time and publication delay. Why could they have not done this with more papers to get a better estimate? Poor measurement of this variable would also probably bias the estimates downward.*

We echo your concern that our investigation of journal review time is not sophisticated. In order to obtain a more reliable and valid estimated review time (from submission to acceptance), we have randomly selected five papers every year for each journal, whose total observation thus increase the original 400 papers to 2252 papers. After collecting all the review data and averaging them out, we gained results as shown by the graph below, which are consistent with the review time previously reported. Most of the submitted papers went through a review processes of between one and two years, with over 95 percent of the submitted papers being accepted within two years.

**Figure R2:** Histogram of Review Time of Randomly Selected APA journals (2252 papers)

**Reviewer 2**

*This paper sets out to examine how time pressure to maintain a fixed periodical quota for journal publications can influence a journal editor’s decision to accept or reject a paper. The authors find that a 10% increase in publication backlog, is correlated with a 0.8% increase in the subsequent rejection rates of new submissions. This seems very intuitive: when an editor has more papers to choose from, (s)he will reject a higher percentage of submissions. This finding seems to be very much what one would expect. I doubt whether this is a very interesting finding to publish.*

Thank you so much for your comment. Although it may appear intuitively obvious, we still believe that this study makes several contributions. A number of researchers have suspected that publication outcome can be affected by the backlog of papers, but there is only one empirical paper, to our knowledge, which has tested this backlog impact, and that in an indirect manner. Although academic journals have differing amounts of space available for accepted papers, the study mentioned argued that there is no evidence showing that journal rejection rates are influenced by their space shortages. We are skeptical about this conclusion because, as the authors acknowledged, the challenge of measuring space shortage caused them simply to use the number of papers submitted to a journal as a proxy for its space shortage, which is then problematically correlated with rejection rates. We believe that this is a very first paper to *empirically* test the effect of the backlog of accepted papers or publication delays on subsequent rejection rates. We show that the journal review process is not as objective as might be hoped for, and that bias is not simply attributed to an editor’s personal view or bias but due also to the very nature of journal periodicals with fixed paper quotas.

*Subsequently, the authors suggest (p. 9) ‘that a low publication delay period, may decrease the quality of the journal because the editor may assume a more lenient view of newly submitted manuscripts in order to fill the quota.’ Results in Table 7 indeed show that publication delays have a significant positive effect on impact factor. However, more information should be given about the impact factor in general and how it is measured in this paper to determine whether the authors indeed prove what they claim to prove. What (in general) is the relation between time after publication and number of citations? Is this a normal distribution? If so, papers will have a relatively small effect on the impact factor of a journal in the first year after their publication. After how many years/what period will the effect of the quality of papers be felt in the impact factor of a journal? Also, how many years/months before an article was published, was it submitted? It might take quite some time before the quality of an article submitted in year t, published in year t+a, affects the impact factor of the journal in year t+a+b. For this study: from which years were the impact factors taken? How many years difference is there between the year of impact factor and years used to calculate publication delay?*

Thank you. This is a very important point and we appreciate your comment. Given that quality is not a main outcome for the current study, we did not take a careful approach to this issue. Thus, as suggested, we have now divided time after publication and its separate effect on number of citations, as in the table below. Indeed, the impact factor is shown to be less likely to be influenced by the first year of publication delays and more by the second year.

**Table R1**: The effect of publication delay on impact factor

| VARIABLES | Impact factor |
| --- | --- |
|  |  |
| Publication delay (log, t-1) | 0.07 |
|  | (0.08) |
| Publication delay (log, t-2) | 0.25** |
|  | (0.08) |
| Publication delay (log, t-3) | -0.09 |
|  | (0.07) |
| Publication delay (log, t-4) | 0.01 |
|  | (0.07) |
| Publication delay (log, t-5) | 0.02 |
|  | (0.05) |
| Count of items (log) | 0.02 |
|  | (0.14) |
| Constant | 2.18*** |
|  | (0.57) |
| Journal fixed effect*_j_* | Y |
| Year fixed effect*_t_* | Y |
| Observations | 359 |
| R-squared | 0.08 |
| Number of journal | 49 |

Coefficients are reported with robust standard errors clustered by each journal in parentheses.

All models include journal and year fixed effects. + *p* < 0.10, **p* < 0.05, ***p* < 0.01, ****p* < 0.001

*The relevance of the study mentioned on page 4, i.e. that a task completed near a deadline is of lower quality than one completed well before the deadline, to the study in this paper is not clear and needs to be explained better. The study mentioned on page 4 is about the effect of time pressure on the work someone is performing him/herself. The paper under review is about the effect of time pressure felt by the editor on the quality of accepted work done by others than the editor.*

Your point is valid. We concur with you that the logic behind a task completed near a deadline is of lower quality is not necessarily relevant to our paper, so we dropped this part for our revision.

*Figures 2 and 3: it is not clear to me how to read these figures. This has to be explained better.*

Thank you so much. We added more explanation on Figures 2 and 3 in the revised paper.
